# Investigation of pathogenic mechanisms in multiple colorectal adenoma patients without germline APC or MYH/MUTYH mutations

**DOI:** 10.1038/sj.bjc.6603789

**Published:** 2007-05-15

**Authors:** C Thirlwell, K M Howarth, S Segditsas, G Guerra, H J W Thomas, R K S Phillips, I C Talbot, M Gorman, M R Novelli, O M Sieber, I P M Tomlinson

**Affiliations:** 1 Molecular and Population Genetics Laboratory, London Research Institute, Cancer Research UK, 44, Lincoln's Inn Fields, London WC2A 3PX, UK; 2 Cancer Research UK Colorectal Cancer Unit, St Mark's Hospital, Watford Road, Harrow HA1 3UJ, UK; 3 Department of Histopathology, Rockefeller Building, University College London, London WC1E 6BT, UK; 4 Institute of Cancer, Bart's and the London Medical School, Queen Mary College, London, UK

**Keywords:** multiple colorectal adenoma, APC mutations, MYH/MUTYH mutations

## Abstract

Patients with multiple (5–100) colorectal adenomas (MCRAs) often have no germline mutation in known predisposition genes, but probably have a genetic origin. We collected a set of 25 MCRA patients with no detectable germline mutation in *APC*, *MYH/MUTYH* or the mismatch repair genes. Extracolonic tumours were absent in these cases. No vertical transmission of the MCRA phenotype was found. Based on the precedent of *MYH-*associated polyposis (MAP), we searched for a mutational signature in 241 adenomatous polyps from our MCRA cases. Somatic mutation frequencies and spectra at *APC,* K-ras and *BRAF* were, however, similar to those in sporadic colorectal adenomas. Our data suggest that the genetic pathway of tumorigenesis in the MCRA patients' tumours is very similar to the classical pathway in sporadic adenomas. In sharp contrast to MAP tumours, we did not find evidence of a specific mutational signature in any individual patient or in the overall set of MCRA cases. These results suggest that hypermutation of *APC* does not cause our patients' disease and strongly suggests that MAP is not a paradigm for the remaining MCRA patients. Our MCRA patients' colons showed no evidence of microadenomas, unlike in MAP and familial adenomatous polyposis (FAP). However, nuclear *β*-catenin expression was significantly greater in MCRA patients' tumours than in sporadic adenomas. We suggest that, at least in some cases, the MCRA phenotype results from germline variation that acts subsequent to tumour initiation, perhaps by causing more rapid or more likely progression from microadenoma to macroadenoma.

Individuals who develop multiple colorectal adenomas (MCRAs) are a genetically and clinically heterogeneous group who are at increased risk of colorectal cancer (CRC). The MCRA phenotype can usefully, but arbitrarily, be defined as 5–100 adenomas during an individual's lifetime. Germline mutations in the *APC* gene account for about 15% of cases with an MCRA phenotype and such patients are classified as having the dominantly inherited condition, attenuated FAP (AFAP) (Spirio *et al*, 1999). An additional ∼35% of such patients have biallelic germline mutations in the MutY-homologue (*MUTYH*, *MYH*) gene and are classified as having the recessive condition MYH-associated polyposis (MAP) (Sampson *et al*, 2003; Sieber *et al*, 2003). MYH deficiency causes colorectal adenomas through somatic hypermutation of the *APC* and K-ras genes. The remaining MCRA patients have no known underlying cause for their phenotype.

*MYH* was found to be a MCRA predisposition gene because a striking excess of somatic G:C>T:A transversion was observed incidentally during an analysis of the *APC* gene (Al-Tassan *et al*, 2002). A similar spectrum of K-ras mutations is also found in MAP patients (Lipton *et al*, 2003a). In another Mendelian bowel tumour syndrome, hereditary nonpolyposis colon cancer (HNPCC), the effects of DNA mismatch repair (MMR) deficiency can be detected somatically through the presence of microsatellite instability (MSI) and frameshift mutations in short repeats within genes such as *BAX* and *TGFBR2* (Woodford-Richens *et al*, 2001). It follows that, should some of the remaining MCRA cases be caused by a defect in DNA repair or by some other form of hypermutation, the somatic mutation spectrum will reflect the underlying cause of genomic instability.

The primary tumour predisposition in familial adenomatous polyposis (FAP) and MAP is to colorectal adenomas via *APC* mutation. FAP and MAP patients develop not only macroscopic adenomatous polyps, but also microadenomas (each comprising at most a few dysplastic crypts) within the large bowel (Lipton *et al*, 2003a). This is consistent with an increased rate of adenoma initiation in FAP and MAP. In HNPCC, by contrast, the primary predisposition is probably to carcinoma (or progression from adenoma to carcinoma) and microadenomas are generally absent. The presence or absence of microadenomas in MCRA patients of unknown origins might therefore provide clues about the origins of the disease. A similar line of reasoning can be applied to the observation that (A)FAP and MAP patients frequently develop extracolonic adenomas, especially in the duodenum. The similarity of the MAP and (A)FAP phenotypes suggests that all individuals predisposed to inactivation of *APC* may develop both colorectal and duodenal adenomas. Interestingly, HNPCC patients are at greatly increased relative risk – but low absolute risk – of small bowel cancer, but have very few adenomas at this site.

To address the origins of the MCRA phenotype, we have determined the spectrum of somatic mutations at *APC,* K-ras and *BRAF* in tumours from 25 MCRA patients with no detectable germline mutation in *APC, MYH* or the MMR genes. We have supplemented our molecular analysis with phenotypic data, microadenoma counts and immunohistochemistry for *β*-catenin expression. We have compared our results with data from MAP and (A)FAP patients. Our results help to classify the remaining MCRA cases and provide clues to their genetic origins.

## MATERIALS AND METHODS

### Sample collection and preparation

Twenty-five MCRA patients were recruited through St Mark's Hospital Colorectal Cancer Unit and blood was routinely sampled. Informed consent was obtained before sample collection. Inclusion criteria for the study were between 10 and 100 synchronous or metachronous colorectal adenomas, irrespective of follow-up or (prophylactic) colectomy (see Table 1 for clinical information). A total of 241 formalin-fixed, paraffin-embedded adenomas were collected. Germline mutations in *APC* and *MYH* had been excluded by screening all exons and exon–intron boundaries using fluorescence single-stranded conformational polymorphism (F-SSCP) analysis or denaturing HPLC analysis (details available from authors). In addition, multiplex ligation-dependent probe amplification (MLPA) analysis had been used to screen for germline deletions or duplications of *APC* using the Salsa MLPA kit P043 APC (MRC Holland, Amsterdam, The Netherlands) according to the manufacturer's instructions. Germline mutations in the MMR genes *MSH2*, *MLH1* and *MSH6* had been excluded previously using F-SSCP analysis (data not shown). In support of these findings, no MSI was observed in our patients' adenomas at the markers used for loss of heterozygosity (LOH) analysis (see LOH analysis) and we therefore did not undertake MLPA analysis for the MMR loci. We also collected 56 formalin-fixed, paraffin-embedded sporadic adenomas (patients with one or two adenomas and no reported family history) from 29 individuals. Existing sets of adenomas from AFAP and MAP patients were largely available for comparison (Lamlum *et al*, 2000; Sieber *et al*, 2003, 2006) although a small amount of extra data was collected for this study. For all patients, DNA was extracted from peripheral blood using standard methods. Tumour and normal tissues were dissected from sections cut from archival, paraffin-embedded specimens and we estimated that the resulting tumour DNAs were derived from at least 70% dysplastic cells. DNA was extracted from these by standard proteinase K digestion at 55°C.

### Mutation detection

F-SSCP analysis was used to screen formalin-fixed, paraffin-embedded tumours for somatic *APC* mutations between codons 1220 and 1620, approximately corresponding to the mutation cluster region and covering the region between the first 20 amino acid *β*-catenin binding/degradation repeat (20AAR) and the first SAMP repeat. The region was subdivided into 12 amplicons (details available from authors). Any sample with a bandshift was sequenced directly in both orientations from a new PCR product. Exon 1 of K-ras, exon 3 of *β*-catenin and the mutational hotspot at codon 600 of *BRAF* were screened by direct DNA sequencing in both orientations. Novel mutations were identified through comparison with the COSMIC database (http://www.sanger.ac.uk/geneti cs/CGP/cosmic) and the European APC database (Laurent-Puig *et al*, 1998).

### LOH analysis

LOH at *APC* was analysed using microsatellite markers just distal to the locus (D5S346, D5S421 and D5S656) on the ABI 3100 sequencer. Constitutionally homozygous markers (and occasional PCR failures) were scored as noninformative. At each marker, LOH was considered present if the area under one allelic peak in the adenoma was less than 0.5 times or greater than two times that of the other allele, after correcting for the relative allelic areas using constitutional DNA. In the infrequent event of discordance between markers, precedence was given to the marker closest to the *APC* gene.

### Immunohistochemistry

Paraffin-embedded sections (4 *μ*m) were dewaxed and rehydrated using a standard xylene method. Endogenous peroxidase was blocked by incubating the slides in 0.3% hydrogen peroxide (H_2_O_2_; BDH Chemicals Ltd., Poole, Dorset, UK)/phosphate-buffered saline (PBS) for 30 min. The slides were incubated in normal rabbit serum (NGS–1 : 25; DAKO Ltd., Ely, Cambridgeshire, UK) for 10 min to block nonspecific binding. Primary antibody (*β*-catenin; Transduction Labs, BD Biosciences, Oxford, UK) was added to the slides at 1 : 100 dilution and incubated for 45 min. Positive and negative control slides were included in every run. The slides were then washed in PBS and incubated with secondary antibody (biotinylated rabbit anti-mouse Ig; DAKO Ltd.) for 35 min. The slides were incubated for 30 min at room temperature, washed in PBS and then incubated with streptavidin–peroxidase conjugate (1 : 500, P397; DAKO Ltd.) for 35 min at room temperature. The peroxidase activity was demonstrated by activated 3,3′-diaminobenzidinetetrahydrochloride solution (Sigma D5637, Poole, Dorset, UK) and 0.1% H_2_O_2_. The slides were counterstained in Cole's haematoxylin (Pioneer Research Chemicals Ltd., Colchester, Essex, UK), dehydrated and mounted using Pertex mountant (CellPath, Newton, Powys, UK). Immunohistochemistry was performed on the multiple adenoma group, sporadic adenomas and, for comparison, a set of patients with FAP. Slides were then examined using a conventional light microscope and the level of expression of *β*-catenin (1, 2 or 3) was recorded separately for the nucleus, cytoplasm and cell membrane according to the intensity of staining in five random high-powered fields per slide. This was compared directly with staining in normal crypts from each sample. For membranous expression, in order to normalise results across different runs of immunohistochemistry, the abnormal staining for each slide was divided by the membrane staining in normal cells. Normal cytoplasmic and nuclear expression was so weak that such a correction was not required. Immunohistochemistry was scored by CT and KH, under the guidance of a gastrointestinal histopathology specialist (MN); any differences were resolved by consensus.

## RESULTS AND DISCUSSION

### Features of the MCRA patients

The clinical features of the 25 MCRA patients are shown in Table 1. All were of white UK origin. The mean age at presentation was 60 years (median=61, range=44–70). Mean polyp number at presentation was 24 (median=21, range=10–76) and the great majority of polyps were mildly dysplastic tubular or tubulovillous adenomas less than 5 mm in diameter. One patient had CRC at the time of presentation. No patient had upper gastrointestinal adenomas and there were no other notable or consistent extracolonic features. Most MCRA patients were isolated cases, although a few patients had a single sibling affected with multiple adenomas. In no case was there multigenerational inheritance of CRAs. In five patients, CRCs were present in other family members, although none of these individuals had been reported as having adenomas.

Our MAP patients presented at a median age of 57 years (range=54–69), not significantly different from the MCRA patients (*P*=0.476, Wilcoxon test). The AFAP patients (median age=43 years, range=27–68) were significantly younger than the MCRA group (*P*=0.027, Wilcoxon test). We did not undertake a comparison of polyp numbers among the patient groups, since this was determined by our inclusion criteria, but the presence of CRC at presentation was similarly frequent in all three patient types (details not shown).

The family histories and phenotypes of our MCRA cases therefore resembled MAP, except that we found no evidence of a predisposition to extracolonic disease in the MCRA group. If the MCRA cases had, like MAP patients, a tendency to hypermutation of *APC* in the gastrointestinal tract, we might expect a similar spectrum of disease to MAP or (A)FAP. Therefore, it is possible that MCRA results either from hypermutation of *APC* in the large bowel only or from a distinct pathogenic mechanism that does not involve the tendency to inactivate *APC*.

### Somatic mutation and LOH frequencies are similar in MCRA and sporadic adenomas, but different from AFAP and MAP

We detected truncating, somatic *APC* mutations in 49 of 221 (22%) adenomas from the MCRA group (Table 1). Of 190 informative tumours, 32 (17%) showed allelic loss (LOH). Two truncating somatic *APC* changes were detected in two tumours and 11 tumours had a single *APC* mutation and LOH. The *APC* mutation and LOH frequencies were not significantly different from those found in the sporadic adenomas (Table 2); however, the AFAP and MAP tumours had significantly higher frequencies of truncating mutations and lower frequencies of LOH than the MCRA tumours (Table 2). No adenoma was found to have a *β*-catenin mutation.

A total of 12 (12%) K-ras mutations were detected in 97 MCRA tumours analysed; 10 mutations occurred at codon 12 and two mutations at codon 13. All of the changes – G12A, G12D, G12V and G13D – had been reported previously to occur frequently in sporadic adenomas and CRCs. A little surprisingly, nine out of 85 (11%) MCRA adenomas analysed had a *BRAF* mutation (V600E); none of these adenomas had serrated morphology. No tumour harboured both K-ras and *BRAF* mutations. The K-ras and *BRAF* mutation frequencies in the MCRA tumours were not significantly different from those in the sporadic adenomas (five out of 34 (*P*=0.47) and five out of 40 (*P*=0.73), respectively; Fisher's exact test).

The similar frequencies of changes at APC, K-ras and *BRAF* in MCRA and sporadic cases suggest that the genetic pathways of tumorigenesis overlap, at least as regards these three genes. The differences from MAP and AFAP reinforce the possibility that hypermutation of *APC* and/or K-ras does not drive tumorigenesis in MCRA patients.

### No definitive mutational signature in the MCRA patients' tumours

In the MCRA patients' tumours, the total of 51 truncating *APC* mutations (Table 1) comprised 44 frameshifts (small insertions or deletions) and seven nonsense mutations (3 C:G>T:A transitions and four G:C>T:A transversions). Although the proportion of frameshift mutations was higher than in the *APC* mutation database (Laurent-Puig *et al*, 1998), this probably resulted from detection bias in small, archival tumours, which were the only samples available from our patients. We have previously observed decreased mutation detection sensitivity in such samples (Groves *et al*, 2002). In support of this contention, the excess of frameshifts in the MCRA tumours was very similar (*P*=0.44, Fisher's exact test) to that in our sporadic adenomas, which had acquired eight frameshift changes plus one A:T>T:A transition and one G:C>T:A transversion. The *APC* mutation spectrum in the MCRA tumours contrasted with the finding that all 25 truncating mutations in the MAP tumours were G:C>T:A transversions (*P*<0.001, Fisher's exact test).

We then considered whether there was evidence of a ‘mutational signature’, especially whether individual patients had a tendency to frameshift changes, to specific nonsense mutations or to LOH at *APC*. In general, no such tendency was evident (Table 1). However, one patient (no. 21) had acquired frameshift mutations in 13 tumours and no nonsense changes and another (no. 23) had five frameshift changes and no nonsense changes. These frequencies were not significantly different from those expected (*P*>0.1, exact binomial test). It was, however, striking that all changes in the tumours of patient nos. 21 and 23 occurred in a particular region of the *APC* gene, between the second and third 20 amino-acid repeats that are involved in *β*-catenin degradation. Such a spectrum of mutations might be expected in patients with a germline *APC* mutation before codon 1280 (Lamlum *et al*, 1999; Albuquerque *et al*, 2002; Crabtree *et al*, 2003) or with a deficiency in MMR that did not manifest as MSI in adenomas (Wu *et al*, 1999). For these reasons, we rescreened patient nos. 21 and 23 for germline mutations throughout the *APC* and the MMR genes *MLH1, MSH2, MSH6* and *PMS2*. We included normal tissue from around the adenomas in this screen in case the cases were mosaics. However, no pathogenic changes were found in any of the genes analysed.

In the MCRA patients, the K-ras mutations comprised six G:C>A:T, five G:C:>T:A and one G:C>C:G changes. Overall, this mutation spectrum was similar to that of the sporadic adenomas, which had nine G:C>A:T, three G:C>T:A and two T:A>A:T mutations (*P*=0.31, Fisher's exact test). Similar to *APC,* these K-ras mutation spectra contrasted with the sole change, G12C (G:C>T:A), found in MAP tumours. As expected, all of the *BRAF* mutations were T:A>A:T transitions resulting in the V600E change. No adenoma had both K-ras and *BRAF* mutations. There was no association between the presence of *APC* and K-ras or *BRAF* mutations (details not shown).

Overall, these findings strongly suggested that there is no common mutational signature in the MCRA group of patients subject to the limitations of screening large genes, such as *APC* in DNA of relatively poor quantity and quality. Despite these difficulties, the difference from MAP was striking, once again suggesting that there is no specific tendency for hypermutation in our MCRA cases.

### No colorectal microadenomas in MCRA cases

Haematoxylin an eosin-stained sections of large bowel from FAP, AFAP, MAP and multiple adenoma patients were examined by a histopathology specialist (MN) for the presence of microadenomas (dysplastic lesions <1 mm diameter) in otherwise normal large bowel mucosa. Microadenomas were present in 16 out of 19 FAP sections examined, the mean density being 0.22/mm. In AFAP, microadenomas were present in two out of seven sections examined from four patients, with mean density of 0.03/mm. In MAP, microadenomas were present in three out of 16 sections examined from six patients at 0.01/mm. No microadenomas were found on examination of 16 sections from seven MCRA patients. The majority of specimens examined were from colectomies and therefore sporadic adenoma cases could not be assessed, as it is very unusual for colectomy to be undertaken in these patients.

Historically, microadenomas have been said to be pathognomic of FAP, but we have demonstrated that they also occur in AFAP and MAP. Given that some FAP microadenomas harbour somatic mutations at *APC* (Thirlwell, unpublished data), it is possible that they are the earliest detectable lesion, which has been ‘initiated’ by ‘two hits’ at *APC* (germline plus somatic mutation for (A)FAP or two somatic mutations for MAP). The absence of microadenomas in our patients suggests that their cells does not acquire ‘two hits’ at *APC* at an appreciably faster rate than the general population. The mechanism of tumour predisposition in the MCRA cases may therefore involve a stage of tumorigenesis that is post-initiation.

### *β*-Catenin expression is increased in MCRA tumours

**Figure 1:**
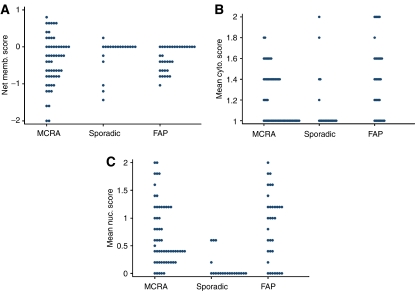
*β*-Catenin expression in adenoma from MCRA and sporadic patients and FAP cases. The dot plots show (**A**) the net mean membranous score, (**B**) the mean cytoplasmic score and (**C**) the mean nuclear score from *β*-catenin immunohistochemistry for MCRA adenomas (series 1), sporadic adenomas (series 2) and FAP adenomas (series 3). Note that, for ease of depiction, the mean scores from individual adenomas are shown as calculated to one decimal place, although for statistical analysis, only mean integer values were used. The plots clearly show the lower nuclear expression in sporadic than MCRA and FAP adenomas. Ideally, a higher mutation pick up rate with less detection bias could be achieved in a larger series of patients resulting in the analysis of a higher number of adenomas.

**Figure 2:**
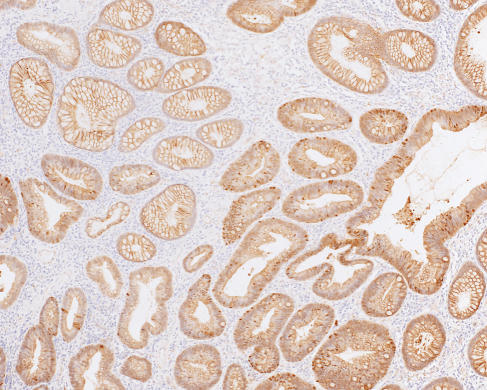
*β*-Catenin immunohistochemistry in MCRA patient's adenoma. Multiple crypts with nuclear *β*-catenin expression are shown.

Relative to the 27 sporadic adenomas, the 57 MCRA tumours showed increased nuclear *β*-catenin expression (*P*=0.0087, Wilcoxon test) (Figure 1). There was, however, no significant difference in nuclear *β*-catenin expression between the MCRA group's tumours and a set of 33 adenomas of similar size from six individuals with FAP (*P*=0.09, Wilcoxon test). Expression was not significantly different between MCRA and sporadic adenomas in the cytoplasm (*P*=0.12, Wilcoxon test) or at the membrane (*P*=0.63, Wilcoxon test) (Figure 2). Logistic regression analysis incorporating the three sites of *β*-catenin expression, adenoma size, degree of dysplasia and patient age found increased nuclear expression to be the only significant variable which distinguished MCRA and sporadic tumours (*P*=0.017, OR=0.22, 95%CI 0.065–0.76).

Unlike the other molecular analyses described above, *β*-catenin immunohistochemistry showed clear differences between MCRA and sporadic adenomas, with expression in MCRA tumours being similar to FAP. The greater Wnt activation in MCRA patients' tumours was too consistent to be readily explained by the inclusion of one or two individuals with cryptic germline *APC* or *MYH* mutations in the MCRA group. Allowing for the fact that colorectal microadenomas probably vary in their progression to macroscopic lesions (Crabtree *et al*, 2001), this finding raises the possibility that increased Wnt activation in MCRA leads microadenomas more rapidly or with greater probability to progress to macroadenomas. Studies to date (Lipton *et al*, 2003b) have not identified germline mutations in Wnt pathway genes in MCRA cases, but many such genes have not yet been screened.

## CONCLUSION

To date, Mendelian CRC syndromes can be categorised in two ways. First, the primary predisposition may be to polyps or to carcinoma. Second, the primary defect may be in either a ‘gatekeeper gene’ (such as *APC*, *LKB1, SMAD4/MADH4* and *ALK3/BMPR1A*) or a caretaker/DNA repair gene (such as the MMR loci and *MYH/MUTYH*). The set of MCRA patients with no germline *APC* or *MYH* mutations is *a priori* likely to have a strong genetic predisposition, given the phenotypic similarity to MAP and AFAP. In our patients, multigenerational inheritance of multiple adenomas was not found, suggesting recessive or, perhaps, multilocus inheritance. Several possible explanations for the MCRA phenotype exist, and these patients may be a heterogeneous group. Nevertheless, our results have provided clues as to the reasons for the MCRA phenotype. We found somatic mutation frequencies and spectra in MCRA adenomas that were very similar to comparable sporadic lesions, distinct from AFAP and, especially, MAP. No microadenomas were found in the MCRA patients' colons. These results suggest that hypermutation of *APC* does not cause our patients' disease and strongly suggests that MAP is not a paradigm for the remaining MCRA patients. Our preferred model is that those few microadenomas, which do occur spontaneously in MCRA cases, have an increased chance of progressing to a macroadenoma. This notion is supported by our immunohistochemical analysis of *β*-catenin expression. If this is the case, it seems unlikely that the predisposition gene is a ‘gatekeeper’ type of tumour suppressor (since MCRA disease seems to be isolated or recessive) or a ‘caretaker’ (since we have found no evidence of a specific DNA repair defect). We tentatively suggest that our data fit best a modified ‘landscaper’ model of tumorigenesis in which the intra- or extracellular microenvironment of cells is altered to favour tumour progression.

## Figures and Tables

**Table 1:** Clinical details and summary of somatic *APC* mutations in MCRA patients

**ID**	**Age**	**Adenoma no.**	**CRC**	**Adenomas analysed**	**Adenomas with truncating mutations**	**Details of truncating mutations (and LOH)**	**Adenomas with LOH only**
1	75	20	No	11	3	1309 delAAAGA, Q1303X C>T, E1379X G>T	NI
2	66	25	No	11	1	1350 delA	NI
3	60	25	No	14	1	1310 delAA+LOH	1
4	71	14	No	18	1	Q1303X C>T	2
5	61	10	No	4	2	E1379X G>T+LOH, E1379X G>T	0
6	51	26	No	2	1	1354 delTT	NI
7	52	27	No	9	2	1465 delAG+1461 delAA, 1465 delAG	3
8	65	10	No	4	2	G1312X G>T+LOH, 1573 del 5bp	0
9	61	19	No	6	2	1493 delA, 1493 delA	3
10	73	10	No	4	0		0
11	68	23	Yes (68)	4	0		1
12	69	12	No	7	3	1426 delG, 1446 delT, 1490 insC	0
13	67	27	No	18	0		4
14	73	76	No	3	1	1350 delA	NI
15	55	10	No	2	0		0
16	55	17	No	2	1	1259 delAT+1473 delT	0
17	52	50	No	6	2	1411 delT, 1472 delT+LOH	0
18	55	18	No	3	1	1490 insC	0
19	49	15	No	3	0		0
20	66	10	No	3	2	Q1338X C>T+LOH, 1472 insT+LOH	0
21	55	30	No	25	13	1489 insT, 1431 delA, 1489 insC, 1489 insT, 1493 delA, 1472 del55bp, 1488 delTT, 1465 del2bp, 1465 del2bp, 1465 del2bp+LOH, 1465 del2bp, 1465 delG+LOH, 1465 insT	3
22	44	33	No	16	3	1319 delC, 1319 delC, 1466 del2bp	2
23	48	32	No	16	5	1465 delG+LOH, 1491 delT, 1501 delT, 1481 delT, 1500 delA+LOH	1
24	64	50	No	7	2	1465 delAG, 1491 delT	1
25	51	21	No	13	1	1465 delAG+LOH	0

Abbreviations: CRC=colorectal cancer; LOH=loss of heterozygosity; MCRA=multiple colorectal adenoma; NI=not informative.

The table shows: age at diagnosis (years), total number of adenomas developed to time of study, presence of colorectal carcinoma (and age), number of adenomas analysed in study' number of adenomas with any somatic truncating *APC* mutation found, details of mutations found (codon plus specific base change) including LOH if present and number of adenomas with LOH but no truncating mutation found (NI at all markers).

**Table 2:** Comparison between *APC* mutation frequencies in MCRA and other patients

	**MCRA**	**Sporadic**	**AFAP**	**MAP**
Truncating mutation	49/221 (22%)	10/56 (18%)^a^	91/242 (38%)^b^	25/34 (74%)^c^
LOH	32/190 (17%)	5/56 (9%)^d^	19/230 (8%)^e^	1/34 (3%)^f^

Abbreviations: AFAP=attenuated familial adenomatous polyposis; MAP=MYH-associated polyposis; MCRA=multiple colorectal adenoma.

Number of adenomas with mutations out of total successfully analysed (%) are shown, as are numbers of adenomas with LOH at *APC* out of total informative tumours (%). *P* values for comparison of mutation and LOH frequencies with those in the MCRA group using Fisher's exact test are as follows: ^a^*P*=0.59; ^b^*P*<0.001; ^c^*P*<0.001; ^d^*P*=0.20; ^e^*P*=0.01; ^f^*P*=0.022. Data were derived from this study, supplemented by our published work on MAP patients (Lipton *et al*, 2003a) and AFAP (Sieber *et al*, 2006).
